# Adolescent Cholelithiasis: Evolving Challenges and Clinical Implications

**DOI:** 10.7759/cureus.103147

**Published:** 2026-02-07

**Authors:** Zhilin Wang, Ke Wang, Yanguang Sha, Guangming Xu, Guangbin Chen

**Affiliations:** 1 Medicine, Wannan Medical College, Wuhu, CHN; 2 Department of Hepato-Pancreato-Biliary Surgery, The Second People's Hospital of Wuhu, Wuhu, CHN

**Keywords:** adolescent cholelithiasis, biliary sludge, choledocholithiasis, laparoscopic cholecystectomy, obesity, pediatric gallstones, sickle cell disease

## Abstract

Gallstone disease in adolescents is no longer a rare clinical finding and has become an increasingly important concern in pediatric and adolescent healthcare. Shifts in lifestyle, rising rates of obesity, and improved survival of children with chronic conditions such as sickle cell disease have collectively altered the epidemiology and clinical profile of adolescent cholelithiasis. Compared with adult gallstone disease, adolescents exhibit distinct etiological factors, variable clinical presentations, and unique management challenges, particularly with regard to the timing and indications for surgical intervention. This editorial synthesizes current evidence on the epidemiology, pathophysiology, clinical manifestations, diagnostic strategies, and management principles of adolescent cholelithiasis, with an emphasis on practical clinical decision-making. Greater awareness of this evolving condition and the development of evidence-based, age-specific management strategies are essential to optimize outcomes and reduce biliary morbidity in this growing patient population.

## Editorial

Gallstone disease in adolescents, once considered uncommon, has emerged as an increasingly recognized clinical entity in pediatric and adolescent surgery [1]. Parallel to global changes in lifestyle, nutritional patterns, and survival of children with chronic diseases, the epidemiology, etiology, and clinical management of adolescent cholelithiasis have undergone notable shifts [2]. Unlike adult gallstone disease, cholelithiasis in adolescents presents with distinct risk factors, heterogeneous clinical manifestations, and unique management dilemmas. This editorial highlights current challenges and key considerations in adolescent cholelithiasis, with an emphasis on clinical decision-making and future directions.

Epidemiological trends

Recent epidemiological data [3] demonstrate a steady increase in the incidence of gallstone disease among children and adolescents. A retrospective study [1] involving 188 pediatric patients diagnosed between 2002 and 2017 reported a persistent female predominance, with female patients accounting for approximately 76.1% of cases. Notably, although nearly half of affected adolescents exhibit identifiable risk factors, a substantial proportion remain idiopathic, underscoring the multifactorial pathogenesis and incompletely understood disease mechanisms.

In Western populations, the rising prevalence of childhood and adolescent obesity is closely associated with an increasing gallstone burden, suggesting that adolescent cholelithiasis may represent, at least in part, a clinical manifestation of broader metabolic health disturbances [4,5]. These trends highlight the need for heightened clinical awareness and proactive risk assessment in vulnerable populations.

Etiology and risk stratification

From a clinical perspective, categorizing adolescent cholelithiasis into hemolytic and non-hemolytic etiologies remains fundamental, as this distinction directly informs surveillance strategies and therapeutic decision-making.

Hemolytic disorders, particularly sickle cell disease, continue to represent a major cause of pigment stone formation in adolescents [6]. Chronic hemolysis leads to sustained hyperbilirubinemia, predisposing patients to early gallstone development [7]. Retrospective data [8] indicate that the prevalence of gallstones among children with sickle cell anemia approaches 27.5%, emphasizing the importance of routine biliary assessment in this high-risk group.

In contrast, non-hemolytic cholelithiasis accounts for the majority of adolescent cases. Obesity, metabolic dysregulation, and medication exposure, most notably ceftriaxone, are increasingly recognized contributors [3]. Emerging evidence [9] suggests that abnormalities in cholesterol synthesis and intestinal absorption independently contribute to pediatric cholesterol stone formation, reinforcing the concept that adolescent gallstone disease shares key pathophysiological features with adult metabolic cholelithiasis.

A distinct clinical entity, pseudolithiasis, is typically transient and drug-related [10]. Recognition of this condition is critical, as it often resolves with conservative management and does not necessitate surgical intervention [10].

Clinical presentation and diagnostic considerations

The clinical spectrum of adolescent cholelithiasis ranges from incidentally detected asymptomatic stones to severe biliary complications. Biliary colic remains the most common presenting symptom, whereas jaundice is relatively uncommon [11]. Importantly, a considerable proportion of adolescents present with complications such as acute pancreatitis, acute cholecystitis, or choledocholithiasis [12], underscoring the need for timely diagnosis and effective risk stratification.

Abdominal ultrasonography remains the cornerstone of diagnosis owing to its safety, noninvasiveness, and cost-effectiveness, making it suitable for both symptomatic evaluation and screening in high-risk populations [13]. When ultrasonographic findings are inconclusive or common bile duct involvement is suspected, magnetic resonance cholangiopancreatography (MRCP) provides valuable anatomical detail [13]. Endoscopic retrograde cholangiopancreatography (ERCP) continues to play a dual diagnostic and therapeutic role in selected cases of choledocholithiasis [14].

Management strategies: balancing intervention and observation

Management of adolescent cholelithiasis must be individualized, carefully balancing the risks of surgical intervention against the likelihood of disease progression and biliary complications.

For asymptomatic, non-hemolytic gallstones and pseudolithiasis, conservative management with close clinical and ultrasonographic follow-up is generally appropriate [15]. Pharmacologic dissolution therapy may be considered in carefully selected patients (in cholesterol stones); however, its application in adolescents remains limited [16].

In contrast, symptomatic cholelithiasis constitutes a clear indication for surgical intervention. Current consensus [17] suggests that cholecystolithotomy should be considered only under highly restrictive conditions, such as preserved gallbladder function and solitary stones, and must be accompanied by rigorous postoperative surveillance.

International guidelines, including those from the European Association for the Study of the Liver (EASL) [15], recommend laparoscopic cholecystectomy (LC) as the treatment of choice for symptomatic gallstones. Among adolescents with hemolytic disorders, particularly sickle cell disease, the threshold for surgery is lower due to the high incidence of symptoms and biliary complications [18]. Consequently, prophylactic cholecystectomy is often advocated even in asymptomatic patients within this subgroup. In patients with concomitant choledocholithiasis, both laparoscopic common bile duct exploration [19] and and staged ERCP followed by LC [20] have demonstrated efficacy and safety in pediatric practice.

Prevention, follow-up, and future perspectives

Preventive strategies should prioritize the early identification of high-risk adolescents, including those with hemolytic disorders, obesity, prolonged exposure to lithogenic medications, or a family history of gallstone disease [13]. Given the rising incidence of obesity-related cholelithiasis, lifestyle interventions targeting weight management and metabolic health are of particular importance [5].

Special attention should be directed toward biliary sludge, which represents a precursor state with a significant risk of progression to overt gallstone disease [21], especially in children with hemolytic conditions. Accordingly, the development of structured follow-up protocols is essential.

Looking ahead, multicenter prospective studies are needed to refine risk stratification, determine the optimal timing of surgical intervention, and elucidate the long-term metabolic and biliary consequences of cholecystectomy during adolescence. As the prevalence of adolescent cholelithiasis continues to rise, improving awareness and adopting evidence-based management strategies will be critical to optimizing outcomes in this unique patient population.

Conclusions

Adolescent cholelithiasis has emerged as a distinct and increasingly prevalent clinical condition. Its diverse etiologies and heterogeneous presentations necessitate individualized, risk-adapted diagnostic and management strategies that differ fundamentally from those applied in adults. While conservative observation remains appropriate for selected asymptomatic patients, early surgical intervention is generally warranted for symptomatic adolescents and high-risk groups, particularly those with sickle cell disease. LC has proven to be both safe and effective in this age group, including for the management of biliary complications. Future efforts should focus on multicenter prospective research and the development of standardized, age-specific guidelines to optimize risk stratification, intervention timing, and long-term outcomes.
